# Socio-economic, epidemiological and geographic features based on GIS-integrated mapping to identify malarial hotspots

**DOI:** 10.1186/s12936-015-0685-4

**Published:** 2015-05-07

**Authors:** Abdul Qayum, Rakesh Arya, Pawan Kumar, Andrew M Lynn

**Affiliations:** Centre for Biology & Bioinformatics, School of Computational & Integrative Sciences, Jawaharlal Nehru University, New Delhi, India; Indira Gandhi National Forest Academy, Dehradun, India; Centre for the Study of Regional Development, Jawaharlal Nehru University, New Delhi, India; Nepalganj Medical College, Banke, Nepal

**Keywords:** Epidemiology, Geographical features, GIS-integrated mapping, Malarial hotspots, Socio-economics

## Abstract

**Background:**

Malaria is a major health problem in the tropical and subtropical world. In India, 95% of the population resides in malaria endemic regions and it is major public health problem in most parts of the country. The present work has developed malaria maps by integrating socio-economic, epidemiology and geographical dimensions of three eastern districts of Uttar Pradesh, India. The area has been studied in each dimension separately, and later integrated to find a list of vulnerable pockets/villages, called as malarial hotspots.

**Methods:**

The study has been done at village level. Seasonal variation of malaria, comparison of epidemiology indices and progress of the medical facility were studied. Ten independent geographical information system (GIS) maps of socio-economic aspects (population, child population, literacy, and work force participation), epidemiology (annual parasitic index (API) and slides collected and examined) and geographical features (settlement, forest cover, water bodies, rainfall, relative humidity, and temperature) were drawn and studied. These maps were overlaid based on computed weight matrix to find malarial hotspot.

**Results:**

It was found that the studied dimensions were inter-weaving factors for malaria epidemic and closely affected malaria situations as evidenced from the obtained correlation matrix. The regions with water logging, high rainfall and proximity to forest, along with poor socio-economic conditions, are primarily hotspot regions. The work is presented through a series of GIS maps, tables, figures and graphs. A total of 2,054 out of 8,973 villages studied were found to be malarial hotspots and consequently suggestions were made to the concerned government malaria offices.

**Conclusion:**

With developing technology, information tools such as GIS, have captured almost every field of scientific research especially of vector-borne diseases, such as malaria. Malarial mapping enables easy update of information and effortless accessibility of geo-referenced data to policy makers to produce cost-effective measures for malaria control in endemic regions.

**Electronic supplementary material:**

The online version of this article (doi:10.1186/s12936-015-0685-4) contains supplementary material, which is available to authorized users.

## Background

Malaria is a parasitic protozoal disease caused by parasites of *Plasmodium* genus. The parasite belongs to the diverse group of unicellular eukaryotes called protozoa. The genus has 250 *Plasmodium* species, but *Plasmodium falciparum* and *Plasmodium vivax* [1] are two key species found in the Indian sub-region. Falciparum malaria is the most severe form worldwide [2-4], but *P. vivax* is the most important species in the study area of the present work [5]. Malaria is a major health problem in the tropical and subtropical world. Around 2.5 million malaria cases are reported annually from Southeast Asia, of which India alone contributes 76% in malaria incidence [6].

**Figure 1:**
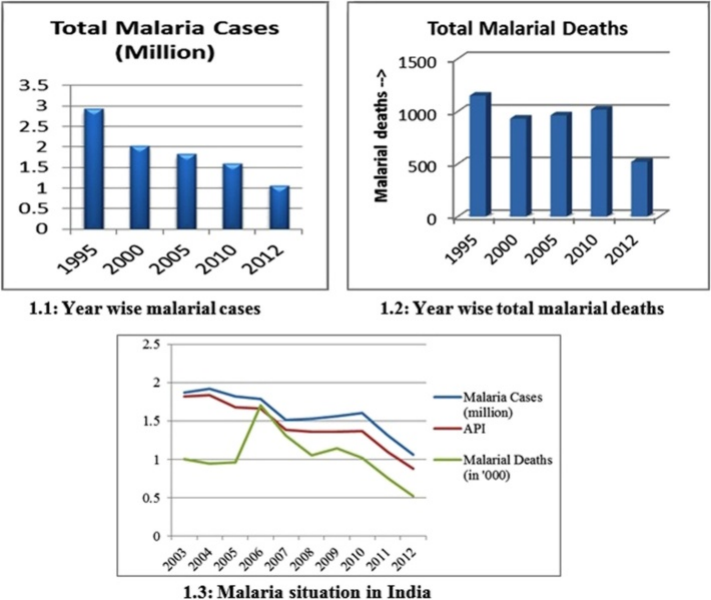
Malaria situation in India and annual deaths. **1.1** Year wise malarial cases. **1.2** Year wise total malarial deaths. **1.3** Malaria situation in India.

Eighty-nine percent of the Indian population resides in malaria-endemic regions. It is a public health problem in most part of the country. Various actions, including passive surveillance of malaria by primary health centres (PHCs), community health centres (CHCs), malaria clinics, use of artemisinin-based combination therapy (ACT), and introduction of intervention such as rapid diagnostic tests (RDTs) for malaria cases, have been taken under the directorship of the National Vector Borne Disease Control Programme (NVBDCP), New Delhi [7]. Mathematical analysis has established the progress and achievement of these action plans (Figure 1 (1.1 and 1.2)). In India, the number of malaria cases reported has decreased from 2.93 million (1995) to 1.08 million (2012), while the number of malarial deaths has decreased from 1,151 (1995) to 519 (2012). From year 2003 to 2012 total malarial cases, annual parasitic index (API) and number of deaths due to malaria has persistently decreased (Figure 1 (1.3)).

Around 27% of Indians live in high-transmission zones where malarial cases are above one per 1,000 persons [7]. Researchers working in the malaria field appreciate that it is a focal disease and the topography of the land is an important consideration in understanding the local epidemiological situation [8]. Such high malaria incidence is primarily because of the drug resistance of its parasites [9]. There are various other reasons, including excessive deforestation [10], indiscriminate use of pesticides in agriculture, demographic shifts, for this enhanced rate of spread of this deadly disease. For vector-borne diseases, factors such as proportion of infectious mosquitoes, vector population density, infecting rates after biting, vicinity of breeding grounds, climatic factors particularly rainfall and relative humidity (RH), are known to have a strong influence on the biology of mosquitoes. To establish seasonal variation and annual variation, geographical information system (GIS) mapping was carried out [11]. In the *terai* region of Eastern Uttar Pradesh the spread of vector-borne diseases has become uncontrolled especially during the rainy seasons [5]**.**

To find malarial hotspot sites, various works was done at macroscopic level by Srivastava *et al.* for tribal states of India [12], by Nath *et al.* for Sonitpur District Assam, by Daasha *et al.* for Koraput District in Orissa [13], by Srivastava *et al.* for Mewat region, Haryana [14], by Agarwal *et al.* for Gwalior City, by Srivastava *et al.* for Kheda District in Gujrat [15], Yadav *et al.* for Udalguri District in Assam. However, much work has to be done by widening the horizon of inclusion of malaria causing factors and there has to be work at village level.

**Figure 2:**
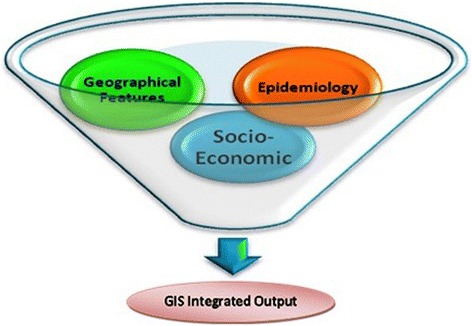
Major segments in the work.

Malaria control action plans are dying out due to improper implementation, inadequate surveillance and lack of geo-referenced information to pinpoint the trouble spots for timely preventive actions [3]. The present work emphasizes the analysing of the malaria epidemic situation and attaches various dimensions of socio-economic situations, epidemiological circumstances and geographic conditions to develop an integrated map based on the application of GIS (Figure 2). GIS has been widely accepted as a mapping device for anti-malarial plants to develop geo-referenced attributes of all such plants with anti-plasmodic actions [16]. Studies have already been done for geographic association with malaria prevalence and have established that a positive correlation for malaria exists with proximity to water bodies [17]. In Huang-Huai, China it was found that 74% of malaria cases were located within 60 m of water bodies and the risk rate among the people living there was significantly higher than elsewhere [18]. The socio-economic data, as well as quantitative and qualitative information on health facilities, have spatial basis and can be integrated [3]. GIS mapping has already been done for the study area [5] at PHC/CHC level but the aim here was to extend this up to the villages. Socio-economic and physico-chemical factors could also be important causes of malaria endemicity in the study region. The data in this work have been acquired from Landsat Thematic Mapper, from Census India 2011 and epidemiological data were collected from district malaria offices (DMO).

**Figure 3:**
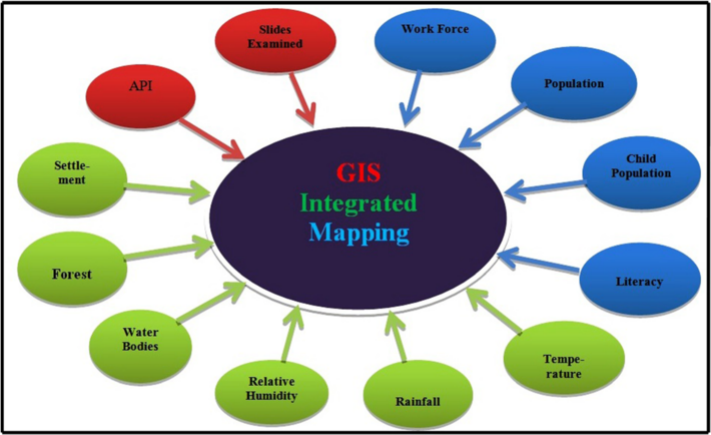
Dimensions in GIS-integrated mapping approach.

With developing technology, the role of tools such as GIS has captured almost every field of scientific research, be it vector-borne diseases [5]**,** forest fire management [11], water harvesting, hydrology, flood prone areas or climate change issues. It has become a principal tool in malarial mapping [2,3,13-15,19,20], and helps with quick retrieval of information and map generation to highlight hotspots of malaria incidence. . Hotspot refers to an area or geographical region of relatively higher importance which is based on parameters such as symptomatic cases and asymptomatic cases. It signifies for the region of focused intervention by the authorities to utilize the limited resources optimally for combating the malaria. The present work is an amalgamation of ten parameters, of which socio-economic (workforce participation (WFP), population, child population and literacy), geographical features (settlement, forest cover, water body, rainfall, RH and temperature) and epidemiology (API and number of slides collected and examined) are the three dimensions (Figures 2 and 3). After overlaying all these parameters, the most vulnerable villages were selected.

The objective of the current study was to develop socio-economic and climatic factors, geographical parameters, and clinical-data based GIS-integrated databank. It includes finding a list of all those villages/pockets (so-called malarial hotspots) where preferential allotments of government anti-malarial policies are required, which is GIS-integrated output based on the factors directly affecting malaria dynamics. These maps will help the authorities in reducing malarial risk in the area and hotspots will help in devising and designing strategic malaria control measures.

## Methods

### Study area

**Figure 4:**
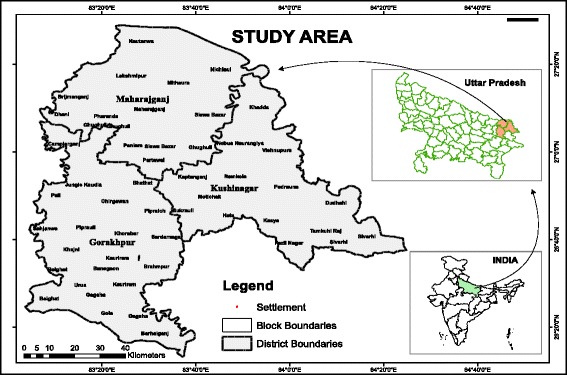
Location of study area.

**Figure 5:**
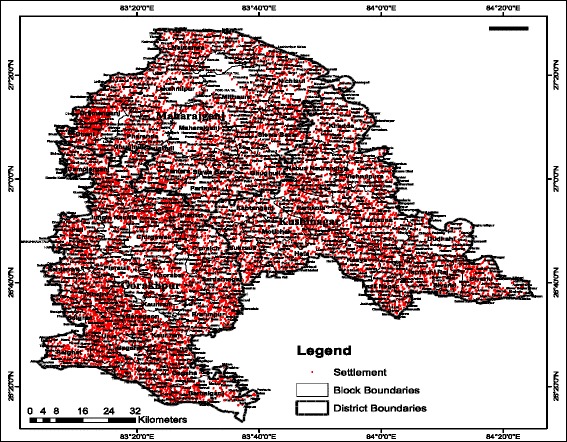
Geographical location of villages of the study area.

The Indian Council of Agricultural Research (ICAR) study area (Figure 4) falls in the agro-ecological sub-region of ‘eastern plain, hot, sub-humid (moist) eco-region’. The area comprises three eastern Uttar Pradesh (UP) districts Gorakhpur (26°13′N to 27°29′N, 83°05′E to 83°56′E and altitude 69 m), Kushinagar (26°39′N to 27°15′N, 83°38′E to 84°15′E and altitude 75 m) and Maharajganj (26°59′N to 27°19′N, 83°09′E to 83°45′E and altitude 66 m). Major soil types found are sandy loam, clay loam and alluvial loam soil, with a total area of 9,291 sq km of which 3.82% is the State area. It lies in the north eastern corner of the most populous state and comprises a large stretch lying to the north of River Rapti, which is a tributary of the Gandak River, and is also surrounded by River Rohini on the northern side, which is its major source of water. There is an international border with Nepal. The study area is a highly dense region of UP State (average population density 1210/sq km) and is home to more than 10.67 million people [21]. It is evident from GIS map (Figure 5) that the villages are settled at a distance of less than a km, densely populated and thus of concern for the malaria epidemic study.

### Socio-economics

**Figure 6:**
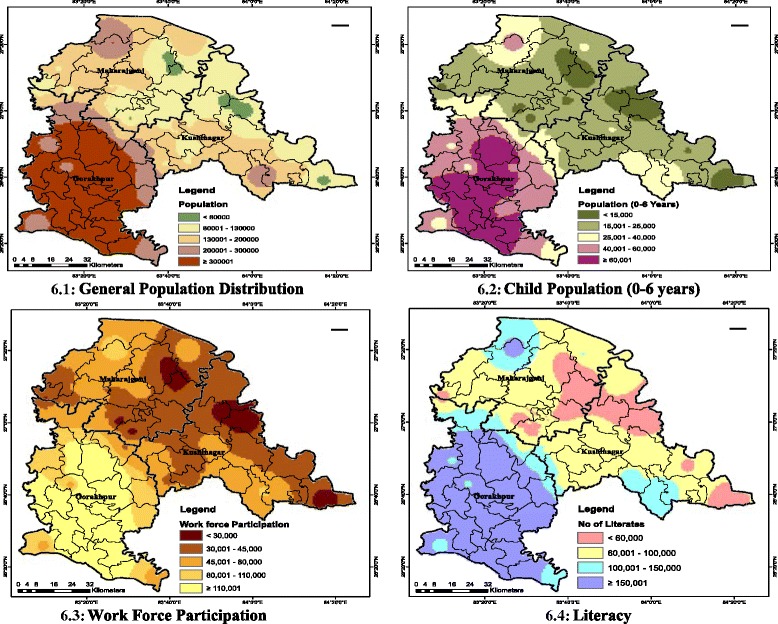
Socio-economic indicator maps. **6.1** General population distribution. **6.2** Child population (up to six years old). **6.3** Work force participation. **6.4** Literacy.

Socio-economic and demographic data are collected based on recent Census 2011 [21] and Economics and Statistics Division, Government of Uttar Pradesh, India. Various factors such as population, income per capita, total household, number of workers, population living below poverty line, etc., (Table 1) are taken into consideration and added as new fields to the spatial databank of the area in ArcGIS 10 environment to generate socio-economic indicator maps (Figure 6) for population (Figure 6 (6.1)), child population (Figure 6 (6.2)), WFP (Figure 6 (6.3)) and literacy (Figure 6 (6.4)).

**Table 1:** **Socio-Economical Features of the study area**

**Agro-Ecological Sub-Regions**	**Eastern Plain, Hot Sub-humid (moist) Eco-Region 13.1, 13.2 & 13.10**	**Total Irrigated Area (‘000 ha)**	**1. Gross irrigated area: 678.6**
			**2. Rain-fed Area: 95.0**
Total Area	917,340 hc	**Land use pattern**	Figure 6-*attached*
Total House Holds^a, d^	1. Rural: 1264192 (87.98%)	**Production of major crops (‘000 tons)**	1. Rice: 964.764
	2. Urban: 172686 (12.02%)		2. Wheat: 1019.363
Health Facility	1. No of PHC: 43	**Livestock/Animal husbandry**	1. Number of cattle: 1,935,250
	2. No of CHC: 15		2. Number of dairy form: 1,033
Population^d^	1. Total: 10,690,142	**Geographical Profile** ^**f**^	1. Average No of days precipitation: 44.88
	2. Male/Female: 5,477,586/5,212,556		2. Average Relative Humidity: 68.33%
	3. Density: 998 People/Sq Km		3. Average high Temperature: 30.92°C
	4. Rural: 8,102,663		4. Average low Temperature: 19.58°C
	5. Urban: 1,049,437		5. Average Mean Temperature: 25.25°C
	6. Rural Population: 88.53%		
	7. Urban/Semi-Urban: 11.47%		
Rainfall (annual)	1. Maharajganj: 1364.1 mm	**Literacy** ^**b**^	1. Male: 3,659,286 (60.24%)
	2. Kushinagar: 1145.1 mm		2. Female: 2,415,006 (39.76%)
	3. Gorakhpur: 1364.1 mm		3. Total: 6,074,292 (52.17%)
Forest Land	1. Area: 56,840 ha	**Area is prone to**	1. Regular: Drought, Pests-Disease
	2. % of total land: 6.20%		2. Others: Flood, cyclone, Hot-cold waves
Work Participation:	1. Total work participation: 3,462,855	**Economy** ^**g**^	1. Agriculture Labors: 494,943
	2. Female total work: 28.3%		2. Main Cultivator: 502,920
	3. Main work participation: 1,708,932		3. Main HH Industry working: 87,400
	4. Female Main Work: 19.1%		4. Monthly Income: 70.3 USD
	5. Marginal Worker: 1,753,923 (16.4%)		5. Rural households: 87.98%

^a:^Calculated on Arithmetic Mean %, ^b:^Calculated on Weighted Mean ^d:^Based on India Census 2011, ^f:^Based on satellite imagery and Climate Research Unit (CRU) UK, ^g:^Economics and Statistics Division, Govt. of Uttar Pradesh, India.

### Epidemiology and clinical data

**Figure 7:**
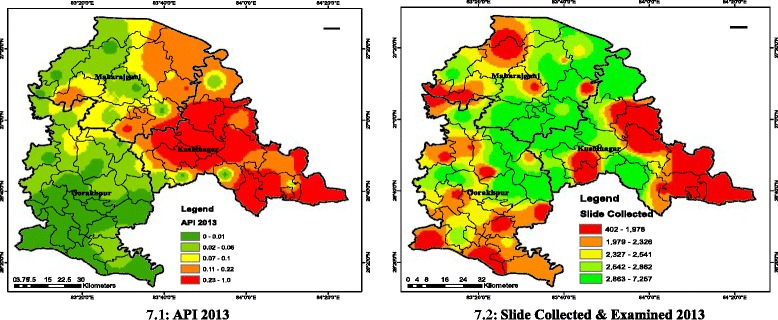
GIS maps for epidemiology. **7.1** API 2013. **7.2** Slide collected and examined 2013.

Epidemiological indices [10] API = (total positive cases for infection/population size) × 1,000 and SPR = (total positive cases/ total slides examined) ×100, are obtained for Kushinagar and Maharajganj and have been plotted to study monthly variation of API for year 2013, while a comparative annual plot of SPR *vis-à-vis* API was done for Gorakhpur. This makes a better picture towards establishing how API and SPR are inter-related. On a monthly basis, malaria incidence in terms of positive cases of *P. vivax* and *P. falciparum* and number of slides examined and collected was obtained from DMO for years 2012 and 2013 at PHC and CHC level. A spatial databank was created in ArcGIS 10 using geo-referenced data of study area obtained through satellite imagery. Inverse distance weightage (IDW) spatial analysis was conducted on clinical data to develop epidemiology maps (Figure 7) including API (Figure 7 (7.1)) and number of slides collected and examined map during year 2013 (Figure 7 (7.2)).

### Geographical features and climatic data

The features conducive for malarial mosquito proliferation, such as water bodies, annual rainfall, RH [22], forest cover, temperature [23], and settlements [24], are collected for the study area using satellite imagery technique. Climate information variable was obtained from the Climate Research Unit (CRU), UK. Shape files of temperature, RH and rainfall were obtained from using ArcMap six discrete GIS maps (Figure 6 (6.1-6.6)).

### Data generation

For the socio-economic parameters, such as population, child population, WFP and literacy, data have been generated at PHC level and settlement map (Figure 5) was produced. The rationale used was to first find percentage settlement in any PHC and percentage settlement of district containing that PHC and then dividing former by latter and multiplying with WFP of that district to generate WFP of PHC. All PHCs, all other districts were similarly calculated for the remaining three socio-economic indicators.

### GIS-integrated mapping

**Figure 8:**
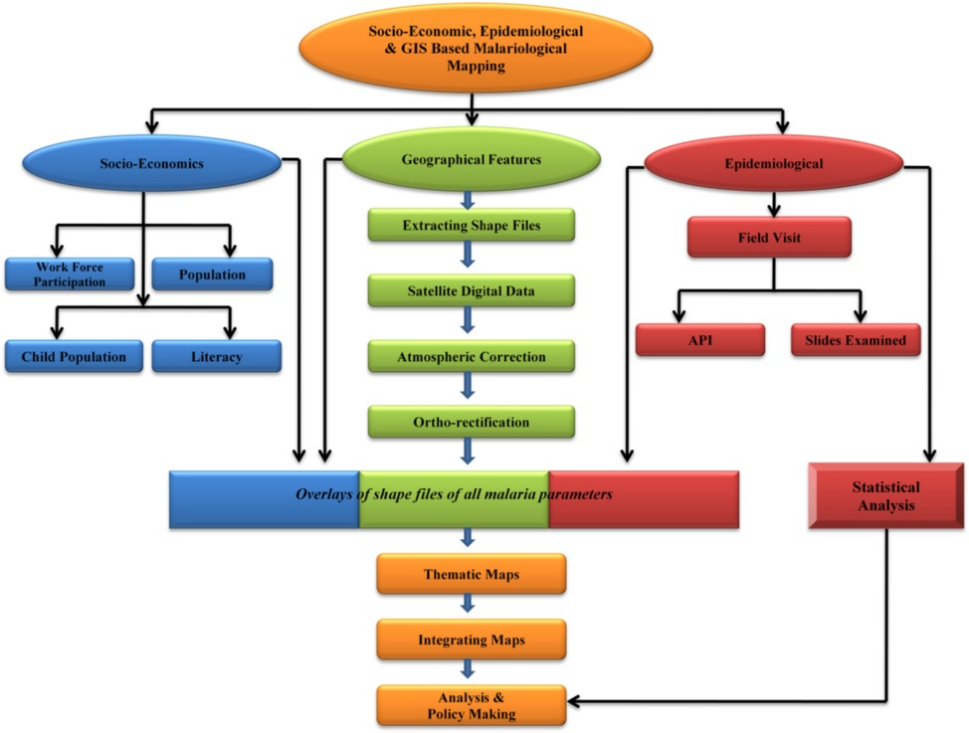
Schematic flowchart: GIS-integrated mapping of socio-economic, geographical features and epidemiology.

A range of geographical features comprising six layers was imported to ArcMap 10 environment. The entire study area, including all CHCs/PHCs, was geo-referenced through numerous GPS coordinates, adjusting the corresponding points in the software environment. Through ArcGIS 10 this set of information was used to develop maps for all the villages in the study area. The analysis was done using ArcMap™ GIS to describe primary risk factors associated with malaria endemicity. Later, the registered sub-centres with their GPS co-ordinates were imported in to ArcGIS environment and spatial data was linked with their attributes. Similarly, various other GIS maps were developed based on socio-economic parameters as well as geographical features as per the schematic flowchart (Figure 8). API value of year 2013 was interpolated using the IDW method to map vulnerable zones in the study area. False colour composite (FCC) imagery (MIR-Red, NIR-Green and Green-Blue) were used to locate general land use in the study area. Vulnerable zones were overlaid on general land to analyse possible malaria causes.

GIS-integrated mapping involves following steps

**Figure 9:**
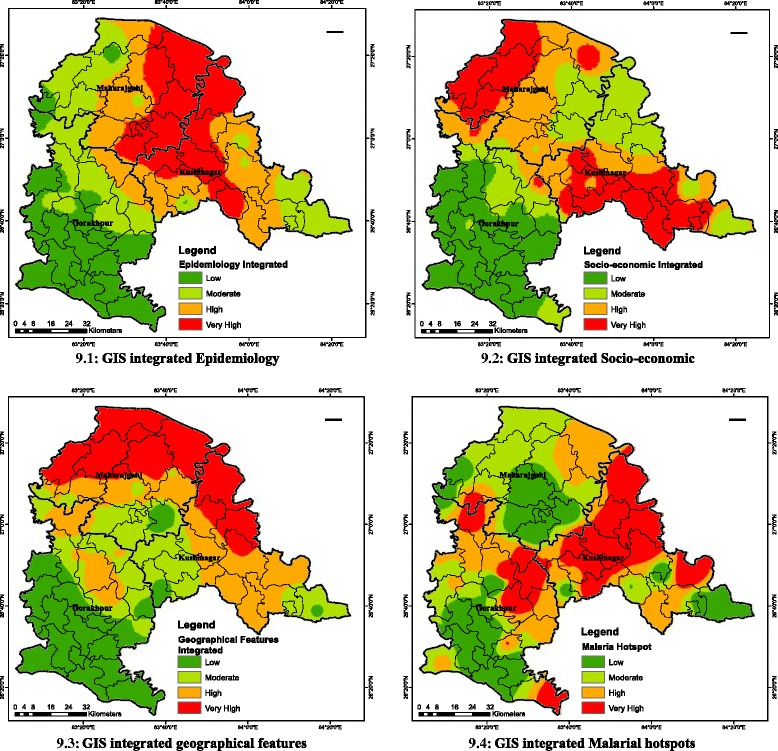
Overlays of epidemiology, socio-economic and geographical features. **9.1** GIS-integrated epidemiology. **9.2** GIS-integrated socio-economic. **9.3** GIS-integrated geographical features. **9.4** GIS-integrated malarial hotspot.

1. GIS layers for 12 malaria factors (Figure 3) were created individually based on natural breaks classes.
2. These individual layers were integrated in to three categories (Figure 9. (91-9.3)) as per Table 2, using standard weights and mathematical equation:
  $$ \begin{array}{l}\mathrm{M}\mathrm{H}\mathrm{S}={\mathrm{HS}}_{\mathrm{se}}\times {\mathrm{HS}}_{\mathrm{e}}\times {\mathrm{HS}}_{\mathrm{gf}}\hfill \\ {}HS={\displaystyle \prod_1^n}n=\left( factor 1\right)\left( factor 2\right)\dots ..\left( factor\;n\right)\hfill \end{array} $$
  where, MHS = Malaria Hotspot, HS_se_ = Hotspot for socio-economic, HS_e_ = Hotspot for epidemiology, HS_gf_ = Hotspot for geographical features.
3. To obtain integrated malarial hotspot (Figure 9 (9.4)), all 12 individual layers are combined using multiplicative function (above equation) and weights and output was categorized using natural breaks. Multiplicative function is used to optimize the respective ranks (Table 2) of each malaria factor or GIS layers.

**Table 2:** **Integrated factors for malaria hotspot identification (Weight Matrix)**

**Factors**	**Standard** ^*^ **weight (%)**	**Class interval** ^**#**^	**Ranks**	**Degrees of Vulnerability**
**Socio-economics**				
**Population**	8	0 – 130,000	1	Low
		130,001 – 300,000	2	Moderate
		300,001 – 450,000	3	High
		450,001 – 1,019,383	4	Very high
**Child Population (0-6 years)**	6	0 – 25,000	1	Low
		25,001 – 60,000	3	High
		60,001 – 154,532	4	Very high
**Work Force Participation**	3	0 – 45,000	4	Very high
		45,001 – 80,000	2	Moderate
		80,001 – 330,209	1	Low
**Literacy**	4	0 – 100,000	4	Very high
		100,001 – 150,000	2	Moderate
		150,001 – 579,2280	1	Low
**Epidemiology**				
**API**	12	0.00 – 0.06	1	Low
		0.07 – 0.10	2	Moderate
		0.11 – 0.22	3	High
		0.23 – 1.05	4	Very high
**Slides Collected & Examined**	8	397 – 2,326	1	Low
		2,327 – 2,541	2	Moderate
		2,542 – 2,862	3	High
		2,863 – 7,203	4	Very high
**Geographical Features**				
**Forest Cover**	11	Non Forested Area	1	Low
		Plantation/Grass lands	2	Moderate
		Wet *Tarai* Swamp	3	High
		Moist deciduous	4	Very high
**Settlements (%)**	5	Low (1.2-1.93)	1	Low
		Moderate (1.94-3.25)	2	Moderate
		High (3.26-8.08)	3	High
		Very high (8.09-38.51)	4	Very high
**Temperature (°C)**	8	23.5 – 25.2	3	High
		25.3 – 25.8	2	Moderate
		25.9 – 26.7	1	Low
**Rainfall (mm)**	13	61.9 – 73.9	1	Moderate
		74.0 – 85.3	2	Very High
		85.4 – 105.4	3	High
		105.5 – 119.7	4	Low
**Water Bodies**	12	Water logged	4	Very high
		River/canals etc	3	High
		Other Regions	1	Low
**Relative Humidity (%)**	10	<60	1	Low
		61-70	3	High
		>70	4	Very High

^*^Based on empirical observations guided by expert’s opinion.

^#^Natural breaks method based.

Weight matrix was used to produce three layers (L_13_, L_14_ and L_15_) initially and later these layers were integrated to produce malaria hotspot (Layer L_16_). The detailed process followed:

1. PHC wise layers of all 12 factors (Figure 2) L_1_, L_2_..... L_12_ was created.
2. All layers were integrated using Boolean operator ‘Union’ to get layer L_13_.
  $$ {\mathrm{L}}_{13}={\displaystyle {\cup}_{i=1}^2\left({\mathrm{B}}_{\mathrm{E}}\right)}\in \mathrm{all}\kern0.5em {\mathrm{L}}_{\mathrm{i}} $$
  B_E_ stands for epidemiology for all layers from L_1_-L_2_
  Thus, layer L_13_ = {B_E_: B_E_ ∈ Layers L_i_; i = 1, 2} (Figure 9 (9.1))
3. Similarly, for Socio-economic factors (B_S_)
  $$ {\mathrm{L}}_{14}={\displaystyle {\cup}_{i=3}^6\left({\mathrm{B}}_{\mathrm{s}}\right)}\in \mathrm{all}\kern0.5em {\mathrm{L}}_{\mathrm{i}} $$
  Thus, layer L_14_ = {B_S_: B_S_ ∈ Layers L_i_; i = 3, 6} (Figure 9 (9.2))
4. And for geographical/climatic factors (B_G_)
  $$ {\mathrm{L}}_{15}={\displaystyle {\cup}_{i=7}^{12}\left({\mathrm{B}}_{\mathrm{G}}\right)}\in \mathrm{all}\kern0.5em {\mathrm{L}}_{\mathrm{i}} $$
  Thus, layer L_15_ = {B_G_: B_G_ ∈ Layers L_i_; i = 7, 12} (Figure 9 (9.3))
5. Malaria hotspot was obtained by integrating layers L_13_, L_14_ and L_15_ using Boolean operator ‘Union’ to get layer L_16_
  $$ {\mathrm{L}}_{16}={\displaystyle {\cup}_{k=13}^{15}\left({\mathrm{B}}_{\mathrm{E},\mathrm{S},\mathrm{G}}\right)}\in \mathrm{all}\kern0.5em {\mathrm{L}}_{\mathrm{k}}; $$
  B_E,S,G_ stand for epidemiology, socio-economic and geographical factors. Thus, required malaria hotspot is Layer L_16_ = {B_E,S,G_: B_E,S,G_ ∈ each layers L_k_; k = 13, 14, 15 (Figure 9 (9.4)).

### Rationale behind weight matrix

It was found in general that malaria incidence was related to land use pattern, water use, higher than average rainfall, greater forest coverage, presence of abandoned water reservoirs, and poor socio-economic status [25]. Weight matrix (Table 2) was constructed based on inputs from experts (Table 3), research findings of related study regions and different regions as well [22,23,25-30], and evidence-based weighting method [26]. Experts were asked to write extent of impact of land-use pattern on all malaria factors in terms of high, moderate, low and negative impact. Evidence-based weighting method was adopted which specifies the malaria relationship with selected factors through weights. However, selected factors were decided based on scrutiny of a series of journals and research articles. Experts suggested vulnerable factors in malaria incidence in response to feedback form. Weight system was derived based on a response of a questionnaire sent to malaria experts familiar with geo-graphics of the study region. The information from journals was combined with the expert opinions based on the relative weighting for a particular malaria factor and its frequency of repetition in various research publications with the suggestion made by the experts. A score out of 100 corresponding to these observations was assigned each malaria factor to constitute weight factors.

**Table 3:** **Correlation matrix**

	**Malarial Hotspot**	**Epidemiology**	**Geographics**	**Socio-Economy**
**Malarial Hotspot**	1.00	0.31	0.31	0.12
**Epidemiology**	0.31	1.00	0.55	0.47
**Geographics**	0.31	0.55	1.00	0.54
**Socio-Economy**	0.12	0.47	0.54	1.00

### Malarial hotspot identification

A malarial risk map was prepared by overlaying ten basic maps (Figure 7 (7.1 and 7.2), Figures 6 (6.1-6.4), 13 (13.1-13.6)). Overlay was done based on the weight matrix. After collecting and evaluating expert response and integrating it with findings of a variety of researches, all the factors were divided into four categories, ranked 1-4. Factors ranked 1 were considered ‘low’, ranked 2 ‘moderate’, ranked 3 ‘high’, and ranked 4 ‘very high’ (Table 2). The cut-off values of categories were decided using natural breaks found within the final data in the study area. Natural Breaks classes were used as it is based on natural groupings inherent in the data. Class breaks are identified that best group similar values and that maximize the differences between classes. The features are divided into classes whose boundaries are set where there are relatively big differences in the data values.

## Results

### Socio-economy

#### Socio-economic and geographical features

Factors such as socio-economic (work participation, economy, urban-rural population, households etc) and geographical features (land cover type, geographical profile, rainfall, forests etc) have good impacts on malaria situation and thus, tabulated (Table 1).

### Land use pattern

**Figure 10:**
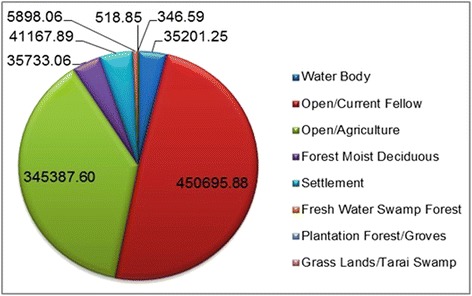
Land use distribution.

The major use of land (Figure 10) is in open/current fallow (49.3%) and agriculture land (37.8%). Most of the land is either for cultivation or is under forest area. Considering available rainfall intensity, the region is good for rice cultivation. Rice fields [31] and forests provide excellent breeding space for mosquitoes. Land use pattern indicates study region could be a malaria potent zone.

### Epidemiology and rainfall

**Figure 11:**
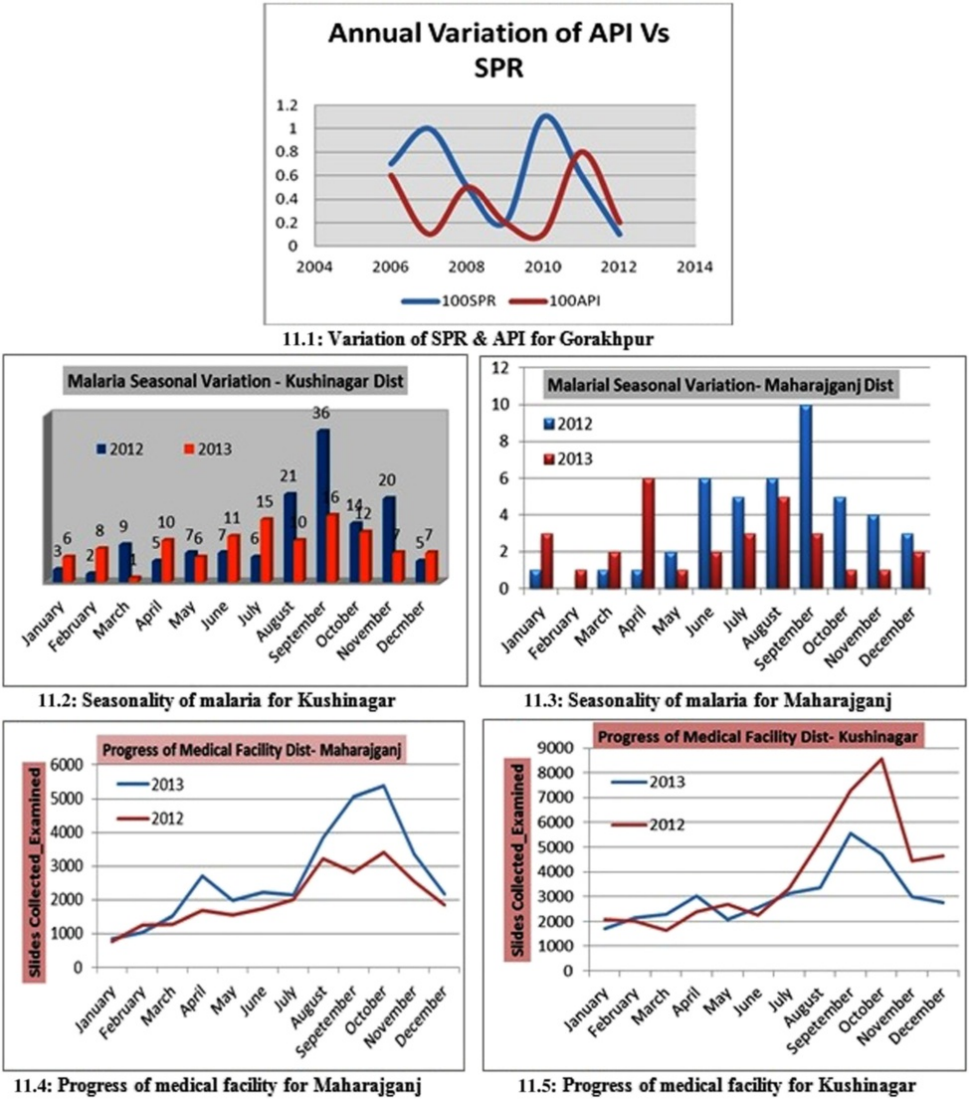
Epidemiology study: Seasonal variation and health facility. **11.1** Variation of SPR and API for Gorakhpur. **11.2** Seasonality of malaria for Kushinagar. **11.3** Seasonality of malaria for Maharajganj. **11.4** Progress of medical facility for Maharajganj. **11.5** Progress of medical facility for Kushinagar.

**Figure 12:**
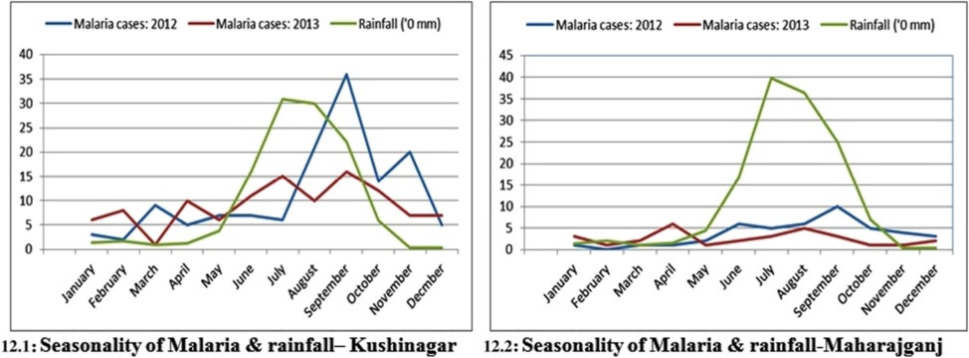
Rainfall vs. malaria cases plot. **12.1** Seasonality of malaria-rainfall, Kushinagar **12.2** Seasonality of malaria-rainfall, Maharajganj.

The study area was bifurcated and the API and SPR for Gorakhpur plotted (Figure 11 (11.1)) to understand its annual co-variation. It was observed that these malarial indices were synchronous in general. For Maharajganj and Kushinagar seasonal variation of malaria incidence, slides collected and examined as well as monthly rainfall was plotted for years 2012 and 2013. It was observed during the rainy seasons (July-October) that malarial incidence was relatively high for both districts in 2012 as well as 2013 (Figure 12 (12.1,12.2)) indicating possible strong correlation of rainfall with malaria. Seasonal variation of malaria during 2012 and 2013 (Figure 11 (11.2, 11.3)) was also similar in general. There was an increase in number of slides collected and examined in 2013 over 2012 and also during rainy seasons more slides were collected and examined (Figure 11 (11.4,11.5)) indicating medical facilities in terms of slides collected and examined had increased to reduce early detection of malaria incidence to reduce malarial deaths.

### GIS based study

#### Land classification

Reveals 8,973 villages/settlement units in the study area (Figure 5). (A list of all village pockets/settlements is provided as Additional file).

### GIS maps for epidemiology

Maps are produced for API 2013 (Figure 7 (7.1)) and health facility indicator in terms of slides collected and examined (Figure 7 (7.2)). These two maps were overlaid to produce an integrated map for epidemiology (Figure 9 (9.1)).

### Socio-economic indicator maps

Four socio-economic elementary maps on general population distribution, child population (up to six years old), WFP and literacy (Figure 5 (5.1-5.4)) were produced. Elementary maps were overlaid in GIS environment to produce an integrated map of socio-economic indicators (Figure 9 (9.2)).

### Geographical indicator maps

**Figure 13:**
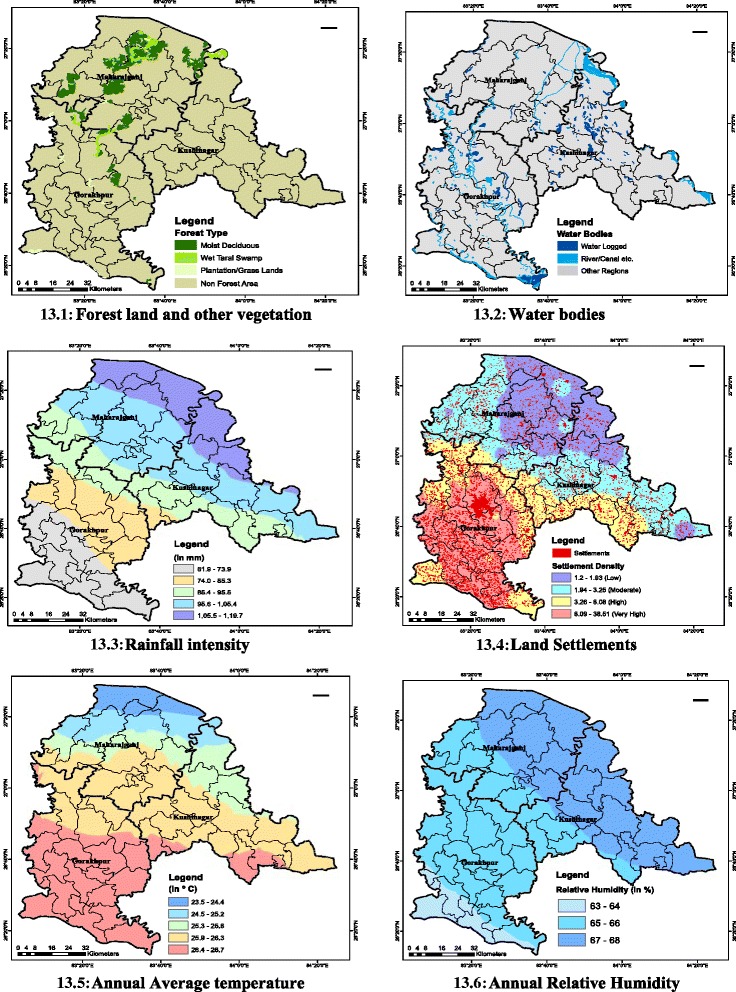
Geographical indicator maps. **13.1** Forest land and other vegetation. **13.2** Water bodies. **13.3** Rainfall intensity. **13.4** Land settlements. **13.5** Annual average temperature. **13.6** Annual relative humidity.

Six elementary maps (Figure 13 (13.1-13.6)) covering major geographical malaria-related factors, including vegetation, water bodies, rainfall, settlements, temperature, and RH were developed to establish possible links between these indicators and malaria. The elementary maps were basic units in developing an GIS-integrated geographical indicator map (Figure 9 (9.3)).

### GIS-integrated maps

To understand spatial distribution of malaria aspects, four layers of socio-economic factors, two layers of epidemiology (clinical) factors and six layers of environment and geographic factors were rated, weighted and ranked (Table 2) on the basis of their importance on malaria incidence. Overlaying of these layers using calculated weights yielded malaria risk map in four classes by natural breaks using ArcGIS 10 software (Figure 9 (9.4)). These classes were very high-risk (2,054), high-risk (2,280), moderate-risk (1,981), and low-risk (2,658). Very high-risk constitutes malarial hotspot and all villages in this class were extracted (Additional file 1).

### Correlation matrix

Matrix was drawn against various malarial factors to find any inter**-**weaving nature and to establish any possible relationship between these parameters (Table 4). The matrix was computed by overlaying layers of malarial hotspot, epidemiology, geographics and socio-economics under the ArcGIS environment. It was observed that epidemiology and geographic features were related to malaria incidence by 55%, socio-economic factors were also largely (54%) related to geographic features, while socio-economics were not a major factor in determining malaria incidence in a given locality; the major factors remain epidemiology and geographic features.

**Table 4:** **Malarial hotspot identification: Classic case of consistent stakeholders and land use pattern**

**Impact of land use pattern on malarial dimensions**												
**Land use**	**Epidemiology**		**Socio-economic factors**				**Geographical features**					
	**E1**	**E2**	**S1**	**S2**	**S3**	**S4**	**G1**	**G2**	**G3**	**G4**	**G5**	**G6**
LU1	0	0	-	-	-	0	+	0	0	0	-	0
LU2	+++	+++	+++	+++	+++	++	++	+++	++	+++	+++	+++
LU3	++	+++	++	+	+	0	+	-	+	-	++	+++
LU4	+++	++	+	++	++	0	+	+	+++	+	+++	-
LU5	+++	++	+++	+++	+++	0	+++	+++	++	+++	+++	+

Extent of Impact: +++ = High, ++ = Medium, + = Low, 0 = None, - = Negative.

LU1 = Barren, LU2 = Settlements, LU3 = *Terai*, grasslands, LU4 = Aquatic Ecosystem, LU5 = Forests/Trees; E1 = API, E2 = Slide examination; S1 = Work Force, S2 = Population, S3 = Child Population, S4 = Literacy; G1 = Temperature, G2 = Rainfall G3 = RH, G4 = Water Bodies, G5 = Forest, G6 = Settlements.

### Malarial hotspot classes

**Figure 14:**
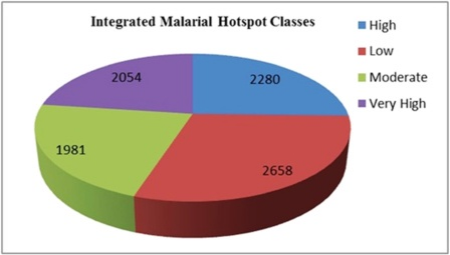
Number of villages in various malarial hotspot classes.

Overlay analysis revealed a total of 2,054 out of 8,973 villages studied were found to be malarial hotspots (Figure 14) and a list of all such villages/pockets is supplied as Additional file 1.

## Discussion

### Socio-economic finding

It is necessary to identify population at risk, their economic level and access to medical facilities for managing an accurate malaria control programme. Since malaria is an environment-dependent disease and hence, by integration of these data with socio-economic and community health levels, it is possible to establish an early warning system for malaria epidemics. The area has 32.4% as total work participation, including 16.4% as marginal worker and has large population below the poverty line (BPL). Literacy level is 52.17% while access to the medical facility is poor, which is the major reason for the poor health recovery due to malarial incidence. The region belongs to low socio economic zones with monthly income ~70.3 USD and 78% population agrarian. The region has 1,436,878 total households in which 87.98% are rural, while 12.02% are urban; the demographic divide lies with 51.25% males, 48.75% females and 3,462,855 works in the entire study area.

### Analysis of epidemiology indices and maps

Malaria incidence in the selected study area is not very prominent if compared with the prevalence in African countries. Instead of API and SPR, 100API and 100SPR was plotted annually for Gorakhpur to highlight numerical values of these epidemiological indices (Figure 11 (11.1)). 100API is algebraic multiplication of API by 100 to magnify the existing API. This is highly useful for the region where API is not so high and magnification eases the study of API variation. These two are plotted on common axis system to find any possible relationship between API and SPR. Theoretically, these are directly related, i.e., ‘sail, swim and sink together’, but observation reveals peculiarity of ‘no proportionate relationship’. However, a major section of the plot is in consistency with the theory and the partial mismatch is because of the error in data collection from the DMO.

Malaria incidence of year 2012 with 2013 was compared and also seasonal and monthly variation of malaria cases for Kushinagar and Maharajganj was plotted (Figure 11 (11.2-11.5)). Epidemiology data for year 2012 was kept to verify the predictive model and results obtained in the study confer with the malaria observed in the villages of ‘very high’ or ‘high’ incidence. GIS mapping for year 2012 for same geographic region was done by and result was compared with the predictive model in the current study. In both districts malaria incidence is relatively high during months July-September (rainy season) in both the years. This establishes positive correlation of malaria cases with rainfall. In rainy seasons the number of breeding sites increases (because of water logging) leading to growth of malaria vectors. However, from 2012 to 2013 there was no significant increase in malaria eradication for the studied area. In general, it remained unchanged and hence the study area demands deeper investigation of the current malaria situation to bring change and satisfactory health achievements.

For Maharajganj, the plot of the number of slides collected and examined for year 2013 showed continuous increase, which is clear indicator of fine discharge of government medical facility. This further indicates the penetration of health facilities to the public. A similar plot for Kushinagar witnessed similar monthly increases in number of slides collected and examined in the same year. This generates a ground for generalization of discharge of health measures for the whole region. Both the districts of the study area could be declared healthy against the health facility available. It is important to note that the medical facility profile of Maharajganj (Figure 11 (11.4)) and Kushinagar (Figure 11 (11.5)) is very similar and is indicative of governmental schemes reinforcing the expansion of the health infrastructure in the study area.

For the microscopic study of malaria incidence in a locality of less critical or malaria-vulnerable areas, a new term of malaria part per million (MPPM) can be introduced and conceptualized (since API for these areas remain in fraction). This is 1,000 times API and serves as numerical convenience for study of malaria of vulnerable localities, after magnifying the obtained API data by 1,000. Although there is no universally accepted definition of ‘malaria vulnerable zones’, it can be noted that for these zones API is low, generally in fraction.

Malarial hotspot identification factors were studied across land use pattern (Table 3). It was found that all types of land use, except barren land, impacts malaria incidence heavily considering epidemiology as one dimension while barren land itself has almost no impact on any of the three dimensions. It was further observed that settlement’s aquatic ecosystems and forest/tree cover had good impact on almost all malaria-affecting factors. Land-use pattern plays crucial role in determining host-vector dynamics.

### Geographical profile

**Figure 15:**
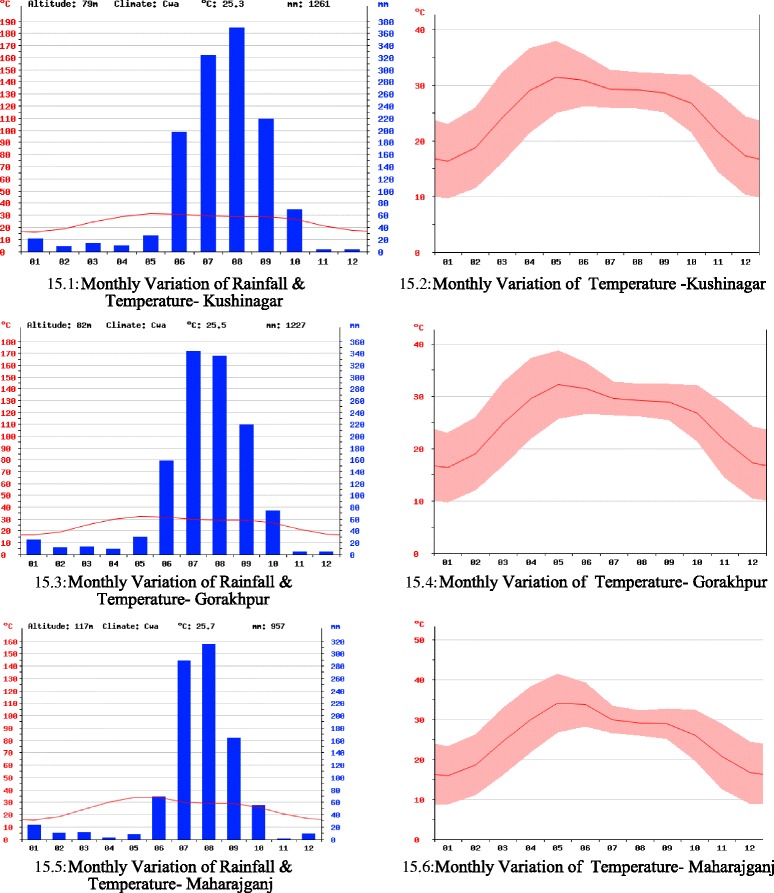
Rainfall and temperature monthly variation. **15.1** Monthly variation of rainfall and temperature - Kushinagar. **15.2** Monthly variation of temperature - Kushinagar. **15.3** Monthly variation of rainfall and temperature- Gorakhpur. **15.4** Monthly variation of temperature - Gorakhpur. **15.5** Monthly variation of rainfall and temperature - Maharajganj. **15.6** Monthly variation of temperature - Maharajganj.

Excess rainfall shows negative correlation [23] with malaria incidence as rain can flush out mosquito larvae [27] and positive correlation with temperature and RH [28]. The map helps identification of breeding places of mosquito larva. It was found that water bodies and forest land nearby human habitation was the main breeding site. Average monthly rainfall and temperature variation were plotted (Figure 15) based on the data obtained from CRU. All the districts reflected similar behaviour (Figure 15 (15.1-15.6)). Temperature bands were plotted with maximum, minimum for each point in a year, which indicates variation of above 12°C in a given day and ranging from 7°C to 41°C.

### GIS analysis

GIS-integrated model possesses well mix of both symptomatic and asymptomatic cases with larger emphasis on the former. For symptomatic cases, slides were collected for patients with malaria symptoms for years 2012 and 2013, and were examined for *P. vivax* and *P. falciparum* positive cases. In GIS-integrated output (Figure 9 (9.4)), a suitable weight based on the matrix (Table 2) is given to both cases to account for developing malaria hotspot. While, indirect parameters including breeding grounds for vectors such as water bodies, high settlement areas and forests; factors for survival of larvae such as rainfall, temperature and RH; other factors such as capacity to afford medical facility indicated through socio-economy parameters, are considered for asymptomatic cases. Under GIS environment, spatial distinction can be easily seen in symptomatic cases (Figure 9 (9.1)) and asymptomatic cases (Figure 9 (9.2 and 9.2)). Moreover, GIS as mapping tool is used to integrate these two cases to bring out malaria hotspot (Figure 9 (9.4)) as key element for early malaria warning system.

Although API of the region falls below the national average, geographical characteristics, proximity to the Himalayan region (major reason for heavy rainfall) and poor socio-economic conditions make the region sensitive to various vector borne diseases. The study region has been epidemic for vector-borne diseases, such as Japanese encephalitis, dengue and chikungunya. It is important to analyse the results to establish control measures against the deadly disease. It was found that the region in the vicinity of Partawal, Fazil Nagar, Motichak, Ramkola and Padrauna PHCs had higher API (Figure 7 (7.1)) over other regions and therefore demands strategic monitoring of government malaria intervention. The number of slides collected and examined was not from the regions of high malaria incidence but from all the regions and were collected uniformly (Figure 7 (7.2)). There must be a spatial shift in slides collection and examination to the region where the API index is high and the additional collection of slides has to be done for these regions of relatively high importance.

PHCs in Gorakhpur are highly populated in comparison to PHCs of other districts. Excess population poses a threat to malaria incidence and hence it possesses relatively higher weight. Regions in the vicinity of wet grass-lands, fresh-water swamp forest and *terai* swampy grass are superior breeding sites for mosquitoes and thus assume more weight, constituting a malaria-sensitive zone. Malaria incidence is likely to be high in eastern Nichlaul, Mithaura and Laxmipur PHC region in times to come.

Water bodies play a pivotal role in malaria dynamics. Vicinity to water bodies is very important for malaria incidence. It varies inversely with distance from the water body (Figure 13 (13.2)). Based on the distance, factor weights are designed (Table 2). Most of the study area falls within cultivable lands. Rice is one of the major crops in the region requiring lots of water which makes a virtual water reservoir and high chance of malaria breeding sites. It was reported that mosquito breeding in rice fields is inversely proportional to the distance from village during a study in Madla District, Madhya Pradesh [31]. However, the precise role of rice fields in maintaining high malaria transmission could not be established but the rice fields contributed significant vector populations and thus high probability of malaria cases is expected.

Moderate rainfall can provide the conditions for breeding of *Anopheles* mosquito and enhances malaria hazard. The soil of Maharajganj and Kushinagar is clay and alluvial loam, which holds water and little additional rain, leads to water logging. Thus, breeding sites are generated and this is the reason that this region has relatively high malaria incidence (Figure 7 (7.1)). It was found that Gorakhpur region has relatively low malaria incidence, the prime reason being heavy rainfall as it is the district with the highest annual rainfall in the whole state. The rainfall flushes out the larvae and excess rainfall possesses lower weight while the moderate (74-95 mm) rainfall that falls in northern Gorakhpur and southern Maharajganj and Kushinagar has high rank and these regions are malaria sensitive with respect to rainfall criteria.

The study area is home to 10,690,142 people with a geographical area of 9,291 sq km and population density of 1,151 per sq km. This amount of land is home to the near equivalent of countries such as Greece, Portugal and Sweden, etc. Land settlement is very dense (Figures 5, 13 (13.4)) making the region highly vulnerable. Gorakhpur City has maximum settlements but almost no malaria incidence is observed, because of better socio-economics and high rainfall.

Southern Gorakhpur City has the maximum of average temperature (averaged annually) among various PHCs/CHCs because of the presence of heavy industry and industrial effluents, while minimum average temperature is found in northern Maharajganj as it has rich forest cover which acts as a sink for warm gases. Temperatures above 32°C have maximum impact on larvae growth but the study region has 26.7°C as maximum average temperature (Figure 13 (13.5)) and thus it is not a major malaria factor, and thus weighted inferiorly.

In the entire study region, RH showed almost no variation (63-68%) and <60% is critical for mosquitoes [22]; thus, it had least impact on vector population and was weighted insignificantly and no significant geographical distinction could be made based on the RH in the study area.

### Health facility hotspots and vulnerable villages

Considering 12-odd factors on the same piece of land for its spatial distribution analysis is the biggest challenge that GIS is capable for. At the same spatial coordinates there might be many contradicting parameters, e.g., forest area, vegetation and rainfall are positive (high rank) factors while population and WFP are negative (less rank) factors. Thus, the weight system (Table 2) has evolved to accommodate two conflicting factors in developing integrated maps towards malarial hotspot identification. Net factor is obtained by weighted multiplication of various malarial factors (Figure 3).

Environmental and climatic factors play a crucial role in influencing malaria incidence and transmission [29]. Sporogenic duration and mosquito survival is highly dependent on temperature. It was claimed that parasitic growth ceases at 16°C or less [30]. Temperatures above 32°C lead to high throughput of vector population. Temperature-induced mosquito deaths occur between 40 and 42°C depending on species [32]. Rainfall does not affect parasites directly but it provides the medium for aquatic mosquito stages and increases RH, which is crucial for mosquito incubation. Monthly average RH below 60% reduces the life of mosquitoes [22]. It was observed that 80 mm average rainfall is crucial for the malarial transmission [30].

## Conclusion

This study could be useful in providing basic knowledge of malaria risk factors and to focus control measures on vulnerable populations alone, thus enabling optimal utilization of resources available, which is essential for developing countries with poor socio-economic indicators. Malarial mapping enables easy update of information and effortless accessibility of geo-referenced data to policy makers to produce cost effective measures for malaria control in endemic regions. The success of such control measures mainly depends on the precise identification and geographical reconnaissance of malarial hotspots. Malaria risks maps are a convenient tool for discussing targeted and cost effective control measures with government authorities. GIS enables the generation of revised maps as soon as new data are available.

Malarial cases in the study region could be attributed to rainfall intensity, temperature, forest cover and humidity as malaria-causing factors, as well as a low socio-economic profile of the population. This study has established that there is a close relationship between socio-economic factors, geographical description, demographic data and epidemiology depiction and malaria incidence. It helps in understanding the malaria transmission pattern based on anthropogenic and environmental factors. Health parameter alone may not be complete and reliable for malaria prediction and thus this integrated approach could be a faultless endeavour to judge malarial hotspots precisely and accurately.

Wide-ranging maps were effective in communicating major findings to the local health authorities, district health administrator and authorities of NVBDCP. With improving socio-economic conditions and deeper penetration of health infrastructure, the present hotspots of malaria may drift and thus GIS mapping becomes much crucial as it offers smooth data updating. As soon as new data are entered, the correct map for the changed scenario is ready, whereas this is a major drawback in the current manual system. The hotspot identification based on GIS mapping could be treated as a priority area for monitoring and surveillance of malaria. It is suggested that a databank of malaria incidence, demographic and socio-economic profile and access to health facilities be established for malaria-endemic regions in the country. Adding these factors to a malaria database will identify hotspots for optimal utilization of resources towards significant malaria control.

### Future work

Using the extrapolation technique for current malaria incidence as well as past, and the hotspot identification used in this study, malaria occurrence could be predicted in future and policy makers could be advised accordingly for effective and optimal distribution of governmental aid for malaria control. Policies need to be streamlined. At present, governmental health aid, such as insecticide-treated mosquito nets and ACT are distributed randomly. These aids have to be distributed in highly targeted fashion, especially when the resources are very limited and need is very high. Similar work has to be extended for the whole land to design a comprehensive governmental plan for developing a ‘Malaria National Map’. The work could be integrated with CSIR, New Delhi’s ongoing bio-prospecting project of open source drug discovery (OSDDs), Malaria Section, to host these malarial maps with a website which is in development phase. It may be further extended to various other vector-borne diseases such as dengue, filaria, chikungunya, kala-azar and Japanese encephalitis to develop similar maps for designing effective control measures against these vector-borne diseases.

## Additional file

Additional file 1:
**List of malaria hotspots on GIS-integrated approach.**

## References

[1] Carlton J, Silva J, Hall N (2005). The genome of model malaria parasites, and comparative genomics. Curr Issues Mol Biol.

[2] Nath MJ, Bora AK, Yadav K, Talukdar PK, Dhiman S, Baruah I (2013). Prioritizing areas for malaria control using geographical information system in Sonitpur district, Assam, India. Public Health.

[3] Yadav K, Nath MJ, Talukdar PK, Saikia PK, Baruah I, Singh L (2012). Malaria risk areas of Udalguri district of Assam, India: a GIS based study. Int J Geogr Inf Sci.

[4] Saxena R, Nagpal BN, Srivastava A, Gupta SK, Dash AP (2009). Application of spatial technology in malaria research & control: some new insights. Indian J Med Res.

[5] Qayum A, Lynn AM, Arya R, Jaiswal SK (2013). GIS integrated epidemiological indices for risk area identification towards malaria control measures. Int J Eng Adv Tech.

[6] WHO (2012). World Malaria Report 2012.

[7] National Vector Borne Disease Control Programme: Malaria situation in India. Delhi, Ministry of Health and Family Welfare, Govt. of India. Available from: http://nvbdcp.gov.in/malaria3.html

[8] Sweeny AW (1998). The Application of GIS in Malaria Control Programs. 10th Colloquium of the Spatial Information Research Centre.

[9] National Vector Borne Disease Control Programme (2009). Guidelines for Diagnosis and Treatment of Malaria in India.

[10] Park K (2013). Textbook of Preventive and Social Medicine.

[11] Jaiswal RK, Mukherjee S, Raju KD, Saxena R (2001). Forest fire risk zone mapping from satellite imagery and GIS. Int J Appl Earth Obs Geoinf.

[12] Srivastava A, Nagpal BN, Joshi PL, Paliwal JC, Dash AP (2009). Identification of malaria hot spots for focused intervention in tribal state of India: a GIS based approach. Int J Health Geog.

[13] Daash A, Srivastava A, Nagpal BN, Saxena R, Gupta SK (2009). Geographical information system (GIS) in decision support to control malaria e a case study of Koraput district in Orissa, India. J Vector Borne Dis.

[14] Qayum A, Lynn A, Arya R (2014). Traditional knowledge system based GIS mapping of antimalarial plants: spatial distribution analysis. J Geogr Inf Syst..

[15] Srivastava A, Nagpal BN, Saxena R, Sharma VP (1999). Geographical information system as a tool to study malaria receptivity in Nadiad Taluka, Kheda district, Gujarat, India. Southeast Asian J Trop Med Pub Health.

[16] Srivastava A, Nagpal BN, Saxena R, Wadhwa TC, Mohan S, Siroha GP (2004). Malaria epidemicity of Mewat region, district Gurgaon, Haryana, India: a GIS based study. Curr Sci.

[17] Van der HW, Konradsen F, Amerasinghe PH, Perera D, Piyaratne MK, Amerasinghe FP (2003). Towards a risk map of malaria for Sri Lanka: the importance of house location relative to vector breeding sites. Int J Epidemiol.

[18] Zhou SS, Zhang SS, Wang JJ, Zheng X, Huang F, Li WD (2012). Spatial correlation between malaria cases and water-bodies in *Anopheles sinensis* dominated areas of Huang-Huai plain. China Parasit Vectors.

[19] Agarwal SA, Sikarwar SS, Sukumaran D (2012). Application of RS & GIS in risk area assessment for mosquito borne diseases- a case study in a part of Gwalior City (M.P.). Int J Advanc Technol Eng Res.

[20] Musa MI, Shohaimi S, Hashim NR, Krishnarajah I (2012). A climate distribution model of malaria transmission in Sudan. Geospat Health.

[21] Census of India (2011). Administrative atlas of India. Office of the Registrar General & Census Commissioner, India.

[22] Pampana E (1969). A Textbook of Malaria Eradication.

[23] Salehi M, Mohammad K, Farahani MM, Zeraati H (2008). Spatial modeling of malaria incidence rates in Sistan and Baluchistan province, Islamic Republic of Iran. Saudi Med J.

[24] Srivastava A, Nagpal BN, Saxena R, Dev V, Subbarao SK (2005). Prediction of *Anopheles minimus* habitat in India- a tool for malaria management. Int J Geogr Inf Sci.

[25] Klinkenberg E, Hoek WVD, Amerasinghe FP (2004). A malaria risk analysis in an irrigated area in Sri Lanka. Acta Trop.

[26] Hanafi-Bojd AA, Vatandoost H, Oshaghi MA, Charrahy Z, Haghdoost AA, Zamani G (2012). Spatial analysis and mapping of malaria risk in an endemic area, south of Iran: A GIS based decision making for planning of control. Acta Trop.

[27] Martens WJ, Niessen LW, Rotmans J, Jetten TH, McMichael AJ (1995). Potential impact of global climate change on malaria risk. Environ Health Persp.

[28] Haghdoost AA, Alexander N, Cox J (2008). Modelling of malaria temporal variations in Iran. Trop Med Int Health.

[29] Cox J, Craig M, Le Sueur D, Sharp B (1999). Mapping malaria risk in the highlands of Africa. MARA/HIMAL Tech Rep.

[30] Adjuik M, Bagayoko M, Binka F, Coetzee M, Cox J, Craig M (1998). Towards an Atlas of Malaria Risk in Africa. First Technical Report of the Mapping Malaria Risk in Africa.

[31] Singh N, Singh OP, Soan V (1989). Mosquito breeding in rice fields and its role in malaria transmission in Mandla district, M.P. Indian J Malariol.

[32] Jepson WF, Moutia A, Courtois C (1947). The malaria problem in Mauritius: the bionomics of Mauritian Anophelines. Bull Entomol Res.

